# Integrated molecular dynamics elucidation of TP53 H179 zinc-binding variants: genomic and structural characterization across NSCLC subtypes

**DOI:** 10.3389/fbinf.2026.1736501

**Published:** 2026-04-10

**Authors:** Ankur Datta, C. George Priya Doss

**Affiliations:** Laboratory of Integrative Genomics, Department of Integrative Biology, School of BioSciences and Technology, Vellore Institute of Technology, Vellore, Tamil Nadu, India

**Keywords:** lung adenocarcinoma, lung squamous cell carcinoma, mutational profiles, TP53, dynamic simulations

## Abstract

**Background:**

Non-Small Cell Lung Cancer (NSCLC), the most prevalent form of pulmonary malignancy, is primarily classified into lung adenocarcinoma (LUAD) and lung squamous cell carcinoma (LUSC). *TP53* gene is the most frequently mutated gene across numerous cancers. p53, a metalloprotein is stabilized by a tetrahedral Zn^2+^ binding motif involving Cys176, Cys238, Cys242, and His179. The His179 site, despite its structural importance, remains underexplored.

**Methods:**

TCGA mutational profiles were evaluated for 616 LUAD and 544 LUSC individuals. This study focuses on mutational perturbations at the His179 locus, a key residue within the protein’s zinc-binding motif. Frequent substitutions at H179 (Y/R/N/L/D) were identified across LUAD and LUSC cohorts. The structural and functional ramifications of these mutations were studied using combinatorial static structural analysis and atomistic molecular dynamics simulations (MDS). Conformational trajectories were analyzed to assess alterations in protein flexibility and functionally critical regions. Binding affinity values of the protein with Zn^2+^ were also evaluated for all mutants.

**Results:**

C > A was the predominant single-nucleotide substitution observed, with *TP53* gene mutations present in 50% of LUAD and 81% of LUSC cases. All five H179 (Y/R/N/L/D) variants exhibited distinct conformational signatures and resulted in compromised protein stability. Contact maps indicated altered residue-level interaction patterns in the mutants as compared to and the wildtype. The energy landscape of the mutants was also observed to be altered in comparison to the wildtype. Structural perturbations were evident in L1 and L2 loops, indicating that these regions are involved in mutation-induced structural plasticity.

**Discussion:**

The results observed underscore the pathogenic potential of His179 mutations within the p53 Zinc-binding motif. The findings highlight the critical role of the Zinc-binding motif in maintaining p53’s conformational fidelity and suggest that specific substitutions may differentially modulate its tumor-suppressive function.

## Graphical Abstract

**Figure:**
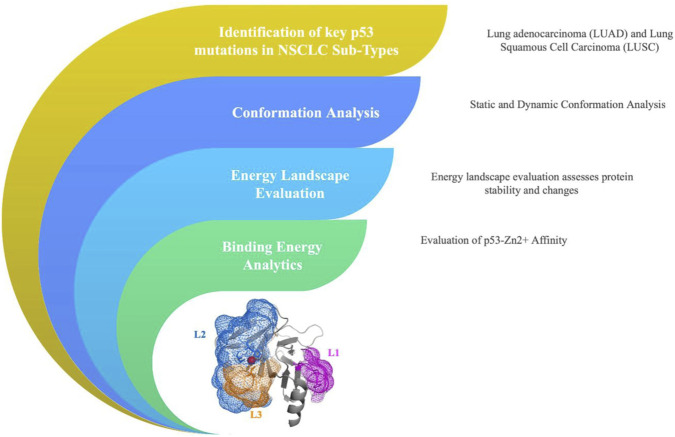

## 1 Introduction

Non-Small Cell Lung Cancer (NSCLC) is the most common cancer affecting the lung tissues and poses significant therapeutic challenges due to its molecular heterogeneity. The two major histological subtypes, lung adenocarcinoma (LUAD) and lung squamous cell carcinoma (LUSC), exhibit distinct etiological and pathological profiles. Epidemiological data highlight that a substantial proportion of cases are diagnosed at late stages, thereby limiting the efficacy of conventional treatment modalities such as chemotherapy, immunotherapy, and targeted therapies (Siegel et al., 2024; Liu et al., 2022). Multiple risk factors contribute to NSCLC pathogenesis, including genetic predisposition, tobacco exposure, and lifestyle influences, with smoking being a significant risk factor for both LUAD and LUSC (Cao et al., 2025; Gao et al., 2009; Schabath and Cote, 2019; Wu et al., 1988). LUAD arises in the peripheral bronchioles and/or alveoli, and LUSC originates in the central region of the lung (Drilon et al., 2012; Gazdar and Minna, 1997). The broad spectrum of clinico-pathological features associated with these subtypes complicates early detection and accurate diagnosis.

Researchers have focused on the mutational profiles of individuals with LUAD and LUSC (Cai et al., 2020; Choi et al., 2017). Genomic profiling has revealed distinct mutational landscapes across LUAD and LUSC, with *TP53* gene emerging as the most frequently altered gene. Comparative analyses report *TP53* gene mutations in approximately ∼90% of LUSC and ∼41% of LUAD cases (Ding et al., 2020). These mutations predominantly cluster within the DNA-binding domain, impairing transcriptional regulation and compromising genomic integrity (Kamaraj and Bogaerts, 2015). *TP53* gene encodes the p53 protein, often termed the “guardian of the genome,” remains a focal point for biomarker development and therapeutic targeting. Curated databases catalog functional consequences of p53 variants (de Andrade et al., 2022). Meanwhile, structural and dynamic modelling has elucidated conformational repertoires of the wildtype monomer (Chillemi et al., 2013). Specific residues, such as Cys176 in the zinc-binding motif, have been highlighted for their role in structural stability (Hephzibah Cathryn & George Priya Doss, 2024).

Mutations in *TP53* gene are strongly associated with repair of damaged DNA, impaired apoptosis, and oncogenic progression (George et al., 2015; Zhou et al., 2019). LUAD exhibits particularly high frequencies of disruptive p53 mutations (Fan et al., 2022). Recent computational frameworks, including deep learning-enhanced MDS, have been employed to classify variants of uncertain significance (Tam et al., 2020; Tam et al., 2021; Tam et al., 2023).

MDS has emerged as a powerful tool to probe local structural perturbations and dynamic behavior, complementing experimental approaches (Koulgi et al., 2013; Pradhan et al., 2019; Wassman et al., 2013). MDS of the behavioral dynamics of the R175H-p53, showcased altered allosteric flexibility in L2/L3 loops, impairing Zn^2+^ coordination (Zhao et al., 2025). Likewise, R273H/C mutants were observed to disrupt the structural integrity of the H2 α-helix, another key region in the DNA-binding domain of the p53 protein (Zhao et al., 2025). The L3 region was also observed to be extended in the R248Q mutant, thereby impairing interactions within the aggregation-prone regions of the p53 protein (Liu et al., 2023). In silico studies of the p53 Y220C mutant emphasized the formation of a narrow crevice on the protein’s surface, which could serve as a potential binding site for small molecules that may help restore the native function of p53 (Xu et al., 2025). An *in vivo* study conducted on mouse cell lines demonstrated the use of anti-p53 (R175H) monoclonal antibodies for accurate diagnosis *via* repeated imaging and autoradiography (Spiegelberg et al., 2025). Protein expression studies have also demonstrated that apo-p53 aggregates faster than holo-p53, thereby highlighting the role of Zn^2+^ in protein aggregation (Wang and Fersht, 2012).

The zinc-binding motif of p53 is critical for its structural integrity and DNA-binding specificity. Zinc coordination typically involves Cys4 or Cys_3_His motifs, with p53 exhibiting a tetrahedral configuration involving Cys176, Cys238, Cys242, and His179 (Kocyła et al., 2021). Approximately 10% of human proteins interact with Zn^2+^, constituting the zinc proteome (Andreini et al., 2006). Each monomer of the p53 tetramer harbors a zinc-binding motif essential for its transcriptional activity. Zinc depletion may not only prevent DNA binding *per se* but also impair sequence-specific recognition, thereby compromising protein native function (Butler and Loh, 2003). Tumorigenic mutations often destabilize the protein by disrupting zinc coordination, altering thermodynamic stability, or both (Blanden et al., 2020). His179, a conserved polar amino acid exposed on the protein surface, plays a crucial role in the zinc-binding motif. Thus, the overall effects of His179 mutants on changes in behavioural dynamics, folding, and compactness of the protein are key aspects that may shed light on the mechanisms underlying the loss of function of the p53 protein.

This study investigates the structural and dynamic consequences of His179 mutations within the p53 zinc-binding motif. By integrating MDS and correlating our findings with the biophysical characteristics of the mutant residues, we aim to delineate how these substitutions affect protein folding, stability, and functional integrity. Such insights will advance mechanistic understanding of p53-driven oncogenesis and inform rational design of therapeutic strategies targeting mutant p53.

## 2 Methodology

### 2.1 Data acquisition and variant profiling

Comprehensive clinical and genomic datasets were accessed from The Cancer Genome Atlas (TCGA) to extract mutational profiles associated with *TP53* gene across NSCLC subtypes, specifically LUAD and LUSC (Colaprico et al., 2016). Comparative analysis of genomic variant distributions revealed 126,254 missense mutations in LUAD and 110,993 in LUSC. *TP53* gene emerged as the most frequently mutated gene in both subtypes. A total of 359 *TP53* gene mutations in LUAD and 254 in LUSC were annotated and compared, yielding 176 shared variants. Among these, five recurrent substitutions were identified at His179, a critical residue in the zinc-binding motif of the p53 protein. p53 functions as a homo-tetramer, with each monomer stabilized by a Zn^2+^ ion coordinated via a Cys_3_His motif involving Cys176, His179, Cys238, and Cys242. The His179 site, despite its structural importance, remains underexplored in NSCLC.

### 2.2 In silico mutational assessment

To evaluate the evolutionary conservation of His179, the ConSurf server was employed under default parameters, assigning conservation scores based on phylogenetic rate variation (Ashkenazy et al., 2016). This metric reflects the degree of purifying selection acting on individual residues. Pathogenicity and mutational stability were further assessed using a suite of predictive tools. Predict-SNP integrates multiple algorithms to classify amino acid substitutions as neutral or deleterious by leveraging evolutionary, physicochemical, and structural features (Bendl et al., 2014). I-Mutant, a support vector machine-based tool, was utilized to predict the thermodynamic stability changes induced by single-point mutations from sequence data (Capriotti et al., 2005). Dynamut was employed to quantify mutation-induced structural perturbations by analyzing vibrational entropy changes and conformational flexibility using graph-based signatures (Rodrigues et al., 2018).

### 2.3 Protein preparation and MDS

For downstream structural and dynamic analyses, the high-resolution X-ray crystallographic structure of p53 (PDB ID: 2OCJ; sequence length: 219) was retrieved from the RCSB Protein Data Bank (Burley et al., 2021). A 3D schematic representation of p53 monomer was prepared using the Pymol visualization tool (Schrödinger and DeLano, 2020).

The CHARMM-GUI web server was utilized to generate the mutation-induced PDB files (Jo et al., 2008). The “manipulate PDB” option in CHARMM-GUI was used to assign the protonation states to the amino acids at the binding sites (C176, H179, C238, and C242) and to solvate the protein at pH 7 using the TIP3P water model. The Monte Carlo method was used to place the Na^+^ and Cl^−^ ions to neutralize the solvated box. A cubic box of size 70 Å was set up to solvate the ions. The NVT ensemble was invoked for equilibration input, and the NPT ensemble at 303.15 K was adopted for dynamics input generation, along with the default CHARMM36m force field. The position of the Zn^2+^ ion with respect to the four interacting protein residues at 176th, 179th, 238th^,^ and 242nd positions was conservatively restrained to a reference bond length of 0.2 nm (r_1_-r_0_ = 0.2 nm) using piecewise harmonic/linear potential energy, as demonstrated in a study carried out by Li et al., 2020; Li et al., 2020). A flat-bottom potential was used between the Zn^2+^ ion and the coordinating residues within the reference bond length. Energy minimization was performed using the steepest descent algorithm at a constant temperature of 303.15K for 50,000 steps. The Particle Mesh Ewald (PME) method was used to calculate the electrostatic interactions. The Verlet cut-off scheme was employed for calculations related to non-bonded interactions, using the default parameters. A V-rescale thermostat and a C-rescale barostat with isotropic coupling type were utilized for the simulations. Cut-off lengths for electrostatic and van der Waals forces were set at 1.2 nm. The LINC algorithm was implemented to constrain hydrogen bonds to their equilibrium lengths during minimization steps and simulation runs (Hess et al., 1997). The simulation’s trajectory frames were saved every 0.1 ns. Independent simulations were performed for each variant over a 500 ns timespan using the GROMACS suite (Abraham et al., 2015; Van Der Spoel et al., 2005). Replicate simulations were conducted for both wild-type and mutant systems to ensure statistical robustness, and root-mean-square deviation (RMSD) standard errors were analyzed across replicates.

### 2.4 Post-simulation conformational analysis

Trajectory data from the primary replicate run, validated to fall within standard error margins (Supplementary Figure S3), were subjected to comprehensive conformational interrogation using GROMACS-integrated modules. Visualization was facilitated through Python libraries Matplotlib and NumPy (Hunter, 2007; Harris et al., 2020). Global structural deviations were quantified *via* backbone RMSD, while root mean square fluctuation (RMSF) analysis provided residue-level insights into local flexibility. Protein compactness and solvent exposure were assessed using radius of gyration (Rg) and solvent-accessible surface area (SASA), calculated with the gmx gyrate and gmx sasa utilities, respectively. To capture distributional trends, kernel density estimates (KDEs) were generated to evaluate the interdependence between Rg and SASA parameters. Principal component analysis (PCA) was performed to project conformational transitions into eigenvector space, enabling the construction of Gibbs free energy landscapes (FEL) for the identification of global maxima and metastable states (Amadei et al., 1993; Onuchic et al., 1997). Finally, binding free energy profiles for each His179 variant were computed using the gmx_MMPBSA suite (Valdés-Tresanco et al., 2021), providing thermodynamic insights into the structural and energetic consequences of His179 substitutions. Statistical comparisons for Zn^2+^ binding based on MMPBSA calculations for triplicate trajectories (6 groups and 18 trajectories), were performed using one-way analysis of variance (ANOVA) followed by Tukey’s *post hoc* test.

## 3 Results

### 3.1 Variant distribution

**FIGURE 1:**
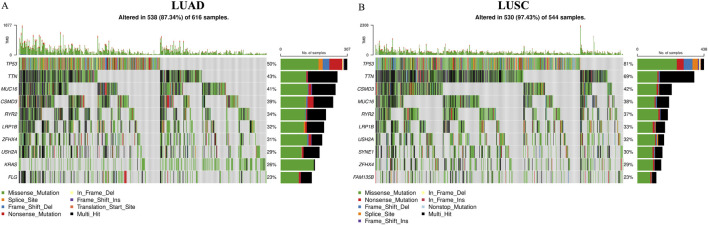
Oncoprint Plot generated using TCGAbiolinks package in R showcasing the mutational distribution in **(A)** Lung Adenocarcinoma (LUAD) patients showcased genomic alterations in 538 (87.34%) of 616 samples; **(B)** Lung Squamous Cell Carcinoma (LUSC) individuals showcased genomic alterations in 530 (97.43%) of 544 samples. The color key for highlighting the various genomic alterations are Missense mutation (Green), Nonsense mutation (Red), Frame Shift Deletion (Blue), Frame Shift Insertion (Violet), Splice Site (Orange), In Frame Deletion (Yellow), Translation Start Site (Orange); Multi Hit alterations (Black).

TCGA is a comprehensive repository of multi-omics data that enables researchers to retrieve and execute biological and clinical workflows. Genomic profiles of 616 LUAD-affected individuals and 544 LUSC-affected patients were retrieved and evaluated. Furthermore, genomic alterations were observed in 87.34% (538 of 616) of LUAD-affected patients and 97.43% (530 of 544) of LUSC-affected individuals, indicating that these alterations significantly contribute to the development of the disease. 50% of 538 (269) LUAD-affected individuals and 81% of 530 (429) patients were observed to harbor a *TP53* gene mutation, indicating that *TP53* gene is the most frequently mutated gene across both forms of cancer. Figure 1 shows the oncoprint, which depicts the distribution of mutations across the top 10 most mutated genes. Figure 1A corresponds to the LUAD affected individuals, and Figure 1B highlights the oncoprint for LUSC subtype. Figures 1A,B also show that missense mutations account for the majority of genomic alterations. Supplementary Figure S1A highlights that 79,895 C > A SNVs were observed for LUAD. Likewise, Supplementary Figure S1B showcases that 52,279 SNVs were observed for C > T and C > A for LUSC. The list of mutations associated with LUAD and LUSC is provided in Supplementary Table S1, with 176 common variants observed in both sub-types. Additionally, the distribution counts of the His179 variants have been listed in Supplementary Table S3 wherein H179R is the most frequently occurring variant (9 cases in both sub-types).

### 3.2 Preliminary mutational analysis

The RCSB PDB was accessed to retrieve the 2OCJ X-ray crystallography structure of the wildtype P53 protein for *Homo sapiens*. The P53 protein structure 2OCJ (sequence length: 219) was assessed for its zinc-binding motif, which was found to be CYS176, HIS179, CYS238, and CYS242. For the current study, variants at position HIS179 were evaluated: H179Y (c.535C>T), H179R (c.536A>G), H179N (c.535C>A), H179L (c.536A>T), and H179D (c.535C>G).

**FIGURE 2:**
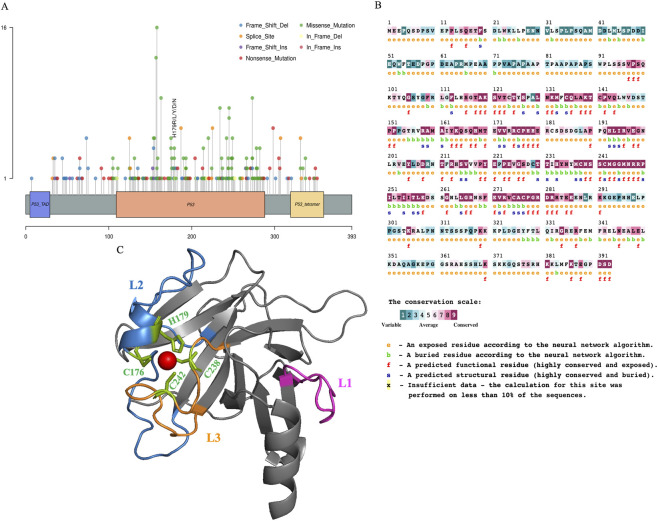
**(A)** Lollipop plot showcasing the mutational distribution for p53 protein; **(B)** ConSurf results indicating the level of sequence conservation for the zinc-binding residue His179, and being exposed to the surface of the protein; **(C)** 3D representation of wildtype p53 protein, wherein L1 (Residues 112–124) region is highlighted in pink, L2 (Residues 163–195) region highlighted in blue, L3 (Residues 236–251) region highlighted in yellow and Zinc in red.

A lollipop plot (Figure 2A) was generated to visualize the frequency and distribution of mutations across all p53 residues. The R175 residue exhibited the highest mutational burden (16 events), while only a single mutation was observed within the trans-activation domain (TAD), alongside multiple sites distributed across the tetrameric complex. Conservation analysis using the Consurf web server (Figure 2B) revealed that His179 is both highly conserved and solvent-exposed, designating it as a predicted functional residue. In contrast, Cys176, Cys238, and Cys242 were also highly conserved but structurally buried, consistent with their stabilizing roles in zinc coordination. Figure 2C showcases the 3D-representation of the wildtype p53 protein, with emphasis on L1 (pink), L2 (blue) and L3 (yellow) loop regions. Zinc is shown in red in Figure 2C.

Pathogenicity of the His179 variants was assessed using Predict-SNP, which integrates outputs from multiple mutational classifiers (MAPP, PhD-SNP, PolyPhen-1, PolyPhen-2, SIFT, and SNAP). Results (Table 1) indicated that all variants were predicted to be deleterious, underscoring their functional relevance. To further evaluate conformational stability, I-Mutant 2.0 and DynaMut analyses were performed (Table 2). Negative ΔΔG values were interpreted as destabilizing effects. Both tools consistently classified H179R, H179N, and H179D as destabilizing mutations, whereas H179Y and H179L yielded contrasting predictions. Notably, H179N exhibited the most pronounced destabilization relative to the wildtype, with ΔΔG values of −1.92 kcal mol^−1^ (I-Mutant 2.0) and −0.254 kcal mol^−1^ (DynaMut) at 303 K, highlighting its significant thermodynamic impact on protein stability.

**TABLE 1:** Results obtained from Predict-SNP webserver.

Wild residue	Position	Target residue	PredictSNP prediction	MAPP prediction	PhD-SNP prediction	PolyPhen-1 prediction	PolyPhen-2 prediction	SIFT prediction	SNAP prediction
H	179	R	DELETERIOUS	DELETERIOUS	DELETERIOUS	DELETERIOUS	DELETERIOUS	DELETERIOUS	DELETERIOUS
H	179	N	DELETERIOUS	DELETERIOUS	DELETERIOUS	DELETERIOUS	DELETERIOUS	DELETERIOUS	DELETERIOUS
H	179	Y	DELETERIOUS	DELETERIOUS	DELETERIOUS	DELETERIOUS	DELETERIOUS	DELETERIOUS	DELETERIOUS
H	179	L	DELETERIOUS	DELETERIOUS	DELETERIOUS	DELETERIOUS	DELETERIOUS	DELETERIOUS	DELETERIOUS
H	179	D	DELETERIOUS	DELETERIOUS	DELETERIOUS	DELETERIOUS	DELETERIOUS	DELETERIOUS	DELETERIOUS

**TABLE 2:** Prediction of stability change for the variants considered for the study.

Position	Variant	I-mutant (DDG change in kcal mol^−1^)	DynaMut (DDG change in kcal mol^−1^)
179	Tyrosine (Y)	0.88 (Increase)	0.031 (Stabilizing)
179	Arginine (R)	−0.28 (Decrease)	−0.292 (Destabilizing)
179	Asparagine (N)	−1.92 (Decrease)	−0.254 (Destabilizing)
179	Leucine (L)	0.95 (Increase)	−0.089 (Destabilizing)
179	Aspartic acid (D)	−0.57 (Decrease)	−0.226 (Destabilizing)

### 3.3 Molecular dynamics simulations

The GROMACS simulation suite was employed to investigate the dynamic behavior of both wildtype and His179 variant forms of the p53 protein over 500 ns trajectories. Multiple structural metrics were extracted to evaluate conformational stability and flexibility. RMSD quantified global deviations from the initial structure (t = 0) across the trajectory, while RMSF provided residue-specific insights into local flexibility.

Statistical analyses of RMSD, Rg, and SASA across triplicate runs are summarized in Table 3. Elevated standard deviations were observed for Run 3 of H179R (0.290 ± 0.133 nm) and H179N (0.329 ± 0.156 nm), as well as Run 2 of H179Y (0.295 ± 0.073 nm) and Run 1 of H179L (0.294 ± 0.070 nm). Notably, the H179L variant in Run 1 exhibited a gradual increase in RMSD before stabilizing, and this trajectory was therefore prioritized for reliable conformational analysis. Distinct deviations were detected at specific timepoints: ∼0.4 nm for H179L (320–340 ns), ∼0.3 nm for H179D (390–410 ns), and ∼0.3 nm for the wildtype protein (450–470 ns).

**TABLE 3:** Results of statistical averages and standard deviations calculated for RMSD, Radius of Gyration and Solvent Accessible Surface Area (SASA) for triplicate trajectory runs.

Variants	RMSD (nm)			Gyration (nm)			SASA (nm^2^)		
	Run 1	Run 2	Run 3	Run 1	Run 2	Run 3	Run 1	Run 2	Run 3
WT	0.241 ± 0.038	0.196 ± 0.027	0.252 ± 0.041	1.673 ± 0.012	1.657 ± 0.010	1.663 ± 0.015	108.87 ± 2.59	108.38 ± 2.47	109.61 ± 2.91
H179Y	0.249 ± 0.040	0.295 ± 0.073	0.287 ± 0.06	1.669 ± 0.010	1.668 ± 0.014	1.671 ± 0.010	110.49 ± 2.61	110.68 ± 3.14	110.59 ± 2.63
H179R	0.24 ± 0.040	0.177 ± 0.046	0.290 ± 0.133	1.672 ± 0.011	1.673 ± 0.008	1.690 ± 0.031	110.63 ± 2.82	109.44 ± 2.61	111.52 ± 3.88
H179N	0.241 ± 0.040	0.218 ± 0.027	0.329 ± 0.156	1.680 ± 0.010	1.661 ± 0.010	1.685 ± 0.033	111.23 ± 2.67	107.97 ± 2.26	110.64 ± 4.30
H179L	0.294 ± 0.070	0.191 ± 0.023	0.204 ± 0.028	1.672 ± 0.016	1.671 ± 0.008	1.673 ± 0.009	107.82 ± 2.82	109.56 ± 2.21	108.70 ± 2.61
H179D	0.231 ± 0.065	0.208 ± 0.0378	0.204 ± 0.031	1.670 ± 0.009	1.659 ± 0.008	1.676 ± 0.010	107.69 ± 2.44	107.87 ± 2.57	110.50 ± 3.00

**FIGURE 3:**
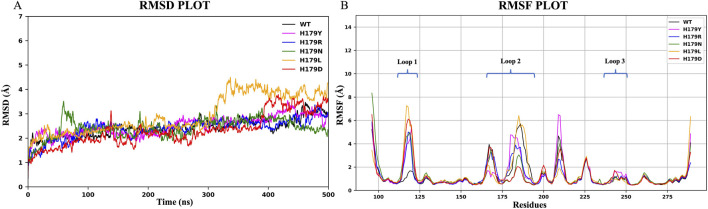
Trajectory plots generated using python libraries **(A)** RMSD plot with deviation depicted in Å on the y-axis and trajectory time-scale depicted in nanoseconds (ns) on the x-axis; **(B)** RMSF Plot showcasing the C-alpha atoms with deviation in Å on the y-axis and residue numbers depicted on the x-axis.

**FIGURE 4:**
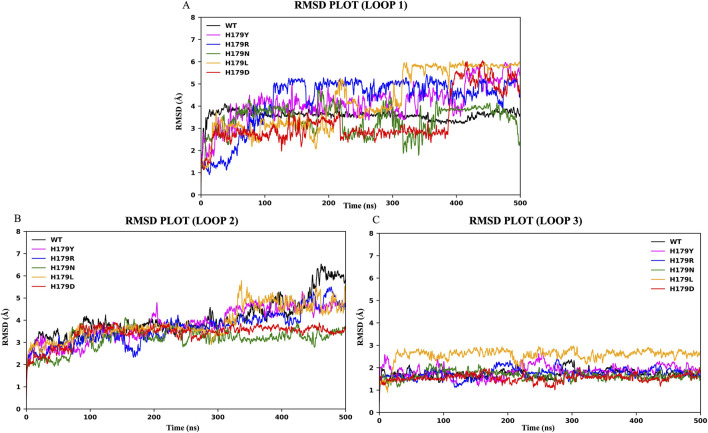
RMSD plots depicted in Å on the y-axis and trajectory time-scale depicted in nanoseconds (ns) on the x-axis for; **(A)** Loop 1 region (Residues 112–124); **(B)** Loop 2 region (Residues 163–195); **(C)** Loop 3 region (Residues 236–251).

RMSD trajectories (Figure 3A) revealed average deviations of 0.241 ± 0.038 nm (wildtype), 0.249 ± 0.040 nm (H179Y), 0.240 ± 0.040 nm (H179R), 0.241 ± 0.040 nm (H179N), 0.294 ± 0.070 nm (H179L), and 0.231 ± 0.065 nm (H179D). Particular attention was given to the Zn^2+^-binding loops: residues Cys176 and His179 in the L2 region, and Cys238 and Cys242 in the L3 region. RMSD plots for these loops are presented in Figures 4B,C. RMSF analysis correlated deviations with Cα atoms of individual residues, highlighting regions of enhanced flexibility (Figure 3B). RMSF results indicated significant deviations among the trajectories, observed for residues 112–124 (L1 region) and 163–195 (L2 region). RMSD trajectories for the L1 region (Figure 4A) revealed average deviations of 0.359 ± 0.027 nm (wildtype), 0.414 ± 0.085 nm (H179Y), 0.427 ± 0.120 nm (H179R), 0.339 ± 0.065 nm (H179N), 0.422 ± 0.135 nm (H179L), and 0.333 ± 0.110 nm (H179D). RMSD trajectories for the L2 region (Figure 4B) revealed average deviations of 0.406 ± 0.087 nm (wildtype), 0.379 ± 0.077 nm (H179Y), 0.364 ± 0.075 nm (H179R), 0.316 ± 0.049 nm (H179N), 0.388 ± 0.075 nm (H179L), and 0.339 ± 0.044 nm (H179D). Minor deviations were observed in the L3 region, which comprises the C238 and C242 zinc-binding motifs, as shown in Table 4. Significant deviations were observed in the L1 and L2 regions. Supplementary Table S2 details the distance between His179 and Zn^2+^ at 100 ns intervals for all variants, derived from the first trajectory run, wherein H179L showcased significant fluctuations in distance throughout the trajectory.

**TABLE 4:** Results showcasing average and standard deviation of RMSD for various loop regions, for Run 1.

Variants	RMSD (nm)		
	Loop 1	Loop 2	Loop 3
WT	0.359 ± 0.027	0.406 ± 0.087	0.172 ± 0.020
H179Y	0.414 ± 0.085	0.379 ± 0.077	0.185 ± 0.027
H179R	0.427 ± 0.120	0.364 ± 0.075	0.174 ± 0.024
H179N	0.339 ± 0.065	0.316 ± 0.049	0.164 ± 0.022
H179L	0.422 ± 0.135	0.388 ± 0.075	0.256 ± 0.032
H179D	0.333 ± 0.110	0.339 ± 0.044	0.154 ± 0.019

**FIGURE 5:**
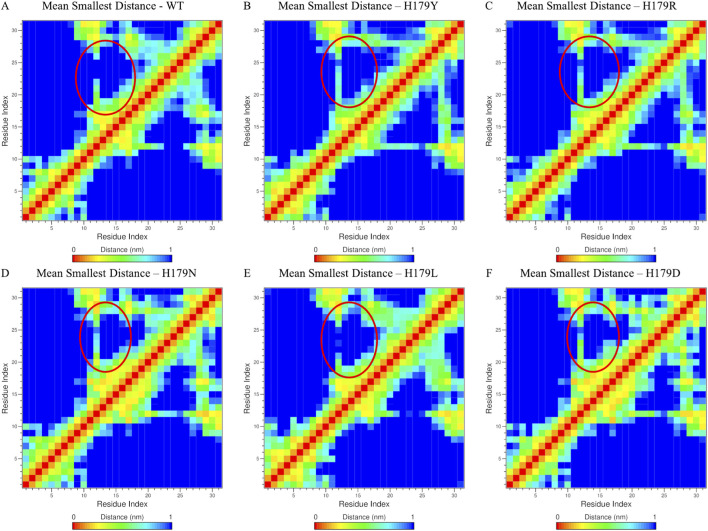
Contact Map residues generated for residues belonging to Loop 2 region for; **(A)** Wildtype; **(B)** H179Y variant; **(C)** H179R variant; **(D)** H179Nvariant; **(E)** H179L variant; **(F)** H179D variant.

Structural snapshots at 100 ns intervals were superimposed to visualize conformational drift (Supplementary Figure S7). Additionally, contact maps for L2 residues were generated to highlight intra-protein interactions contributing to flexibility changes, using a 1 nm distance threshold (Figures 5A–F for wildtype and all variants). Finally, all-atom RMSD plots for the L1, L2, and L3 regions across replicate runs, with standard error estimates, are provided in the Supplementary Figure S4-S6. Structural deviations were visualized from trajectories at 100 ns intervals for all variants and superimposed, as shown in Supplementary Figure S7, thereby indicating the flexible conformations attained at various time points.

### 3.4 Post-MD conformational analysis

**FIGURE 6:**
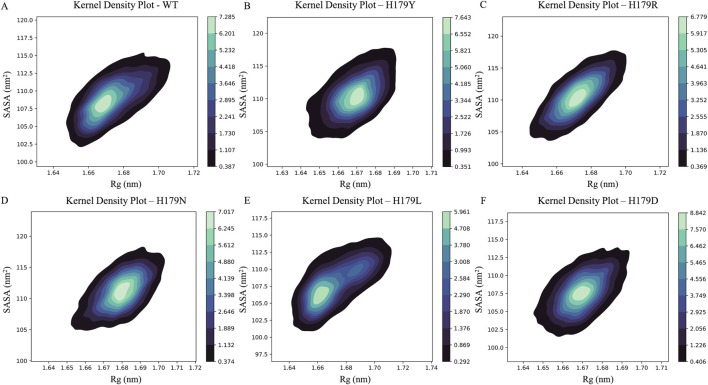
Correlative contour KDE-Plot showcasing association between Rg and SASA for; **(A)** Wildtype; **(B)** H179Y variant; **(C)** H179R variant; **(D)** H179Nvariant; **(E)** H179L variant; **(F)** H179D variant. Association score for wildtype (7.285), H179Y (7.643), H179R (6.779), H179N (7.017), H179L (5.961) and H179D (8.842) variant.

Rg values ranged from ∼1.673 ± 0.012 nm for the wild-type run, ∼1.669 ± 0.010 nm for H179Y, 1.672 ± 0.011 nm for H179R, 1.680 ± 0.010 nm for H179N, 1.672 ± 0.016 nm for H179L and 1.670 ± 0.009 nm for the H179D variant. In the case of SASA, the variation ranged from ∼108.87 ± 2.59 nm^2^ for the wildtype run, ∼110.49 ± 2.61 nm^2^ for H179Y, ∼110.63 ± 2.82 nm^2^ for H179R, ∼111.23 ± 2.67 nm^2^ for H179N, ∼107.82 ± 2.82 nm^2^ for H179L, and ∼107.69 ± 2.44 nm^2^ for the H179D variant. The metrics were plotted as a Kernel Density Plot, shown in Figures 6A–F, representing the protein compactness associated with SASA and flexibility, as determined by the Rg changes. Figures 6A–F highlights the varied distribution of the associative compactness and flexibility of the protein. The association score was observed to be 7.285 for the wildtype, 7.643 for H179Y, 6.779 for H179R, 7.017 for H179N, 5.961 for H179L, and 8.842 for the H179D variant. The Dictionary of Secondary Structure of Proteins (DSSP) module was used to extract secondary-structure information. The results of the DSSP module are presented as a bar plot in Supplementary Figure S2, thereby depicting the percentage of secondary structure elements. The percentage distribution of secondary structure elements is shown in Supplementary Table S4, with variations observed in alpha-helical and beta-sheet regions.

### 3.5 Free energy landscape and zinc-motif binding analytics

**FIGURE 7:**
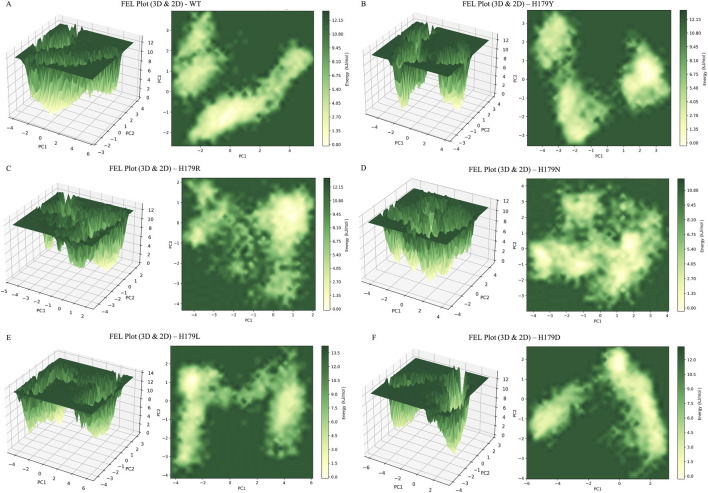
Gibb’s Free Energy Landscape (FEL) Plot represented in 3D and 2D representation for, with their corresponding global energy maxima; **(A)** Wildtype (12.15 kJ mol^-1^); **(B)** H179Y (12.15 kJ mol^-1^) variant; **(C)** H179R (12.15 kJ mol^-1^) variant; **(D)** H179N (10.80 kJ mol^-1^) variant; **(E)** H179L (13.5 kJ mol^-1^) variant; **(F)** H179D (12.0 kJ mol^-1^) variant.

**FIGURE 8:**
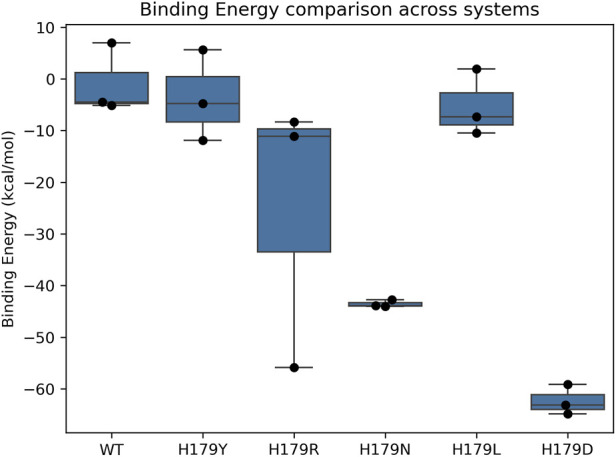
Results obtained from the MMPBSA calculation using the gmx_MMPBSA visualized using python libraries, showcasing the mean of the triplicate trajectories for wild-type and all variants.

The energy component of the conformations throughout the trajectory was determined by projecting the eigenvectors onto the protein’s FEL in the system. Figure 7 comprises a 3D representation of the FEL, showcasing the basin depth. The basin depth is directly proportional to the stability, thereby indicating a stable protein conformation. The top-down view shows the stable states the protein attains along the trajectory. Figures 7A–F also shows the transition states adopted by the p53 protein throughout the trajectory during the MDS. The global energy maxima observed for the protein conformations attained during the trajectory for wildtype, H179Y, and H179R were observed to be 12.15 kJ mol^-1^, for H179N to be 10.80 kJ mol^-1^, H179L to be 13.5 kJ mol^-1^, and H179D to be 12.0 kJ mol^-1^. The binding energy of Zn^2+^ with the protein was also evaluated *via* MMPBSA calculations, and the results of the statistical comparison using one-way ANOVA have been showcased in Figure 8. The results were plotted for all replicate trajectories, and depicts lower binding affinity values for H179N and H179D variants. The results of the ANOVA statistical test have been enlisted in Supplementary Table S5. The metrics observed in the one-way ANOVA statistical test for binding affinity among the six systems (F) = 12.80, p = 0.00018.

## 4 Discussion

The advent of NGS technologies has enabled researchers and clinicians to map and identify alterations in nucleic acid sequences that code for genes (Ostrow et al., 2014; Woo and Li, 2012). Researchers have since used various tools, including MDS, to characterize the trickle-down effect of genomic alterations on the protein’s dynamic behavioral properties (Hollingsworth and Dror, 2018; Karplus and Petsko, 1990). Genetic profiling studies of a Chinese cohort have identified *EGFR*, *TP53*, and *ERBB2* as the most significantly mutated genes (Chen et al., 2020). Researchers have also reviewed the literature and commented on the clinical relevance of *TP53* gene alterations in patients with advanced NSCLC treated with EGFR, ALK, and ROS1 tyrosine kinase inhibitors (Moes-Sosnowska et al., 2024). The findings of our study, as shown in Figures 1A,B, highlight the distribution of genes with the most diverse mutational variation, with the *TP53* gene leading in the number of alterations observed. The DNA-binding domain also harbors a significant percentage of observed variants, and their consequences on structural and behavioral effects have been studied using numerous computational tools (Chitrala and Yeguvapalli, 2014). Further downstream effects of mutated p53 variants have also been reported (Fu et al., 2012, p. 53). R175H is the most frequently observed hotspot mutation across diverse cancer types (Chiang et al., 2021). Researchers have characterized metastasis driver mutations in the *TP53* gene, including H179L (Pandey et al., 2021). A study demonstrated that H179Y-p53 is unable to suppress the growth of H1299 human adenocarcinoma cell lines (Ko et al., 2002). An *in vitro* study by Wrighton et al. (2004) demonstrated increased chemosensitivity of H179L-p53 to cisplatin (Wrighton et al., 2004). However, the impact of the His179 mutations on the zinc-binding motif and the mechanisms underlying the loss of function of p53 caused by His179 variants remains to be studied.

The zinc-binding motif in p53 is tetrahedrally coordinated by Cys-176 and His-179, which belong to the L2 region, and Cys-238 and Cys-242, which belong to the L3 region (Cho et al., 1994). The imidazole ring of the HIS residues bound to Zn^2+^ is a key ligand in the zinc-binding motif (Zhou et al., 2013). His179 possesses a polar side chain with an aromatic imidazole ring, where the nitrogen of the histidine side chain coordinates with the Zn^2+^ ion, which is reported to enhance water affinity (Liao et al., 2013; Song et al., 2022). Cysteine, being one of the other three residues belonging to the tetrahedral zinc binding motif, is hydrophobic and is not exposed to the surface of the protein, depicted in Figure 2C. Earlier reports have evaluated the mechanisms of overall zinc-binding with p53 and other variants, the current study finds novelty in assessing the overall impact of the His179 variants on the core domain and key regions of p53, and also hints towards insights into the loss of native p53 function.

Furthermore, our results, obtained through the ConSurf analysis shown in Figure 2B, highlight that the 179th residue, which is exposed on the protein’s outer surface, is highly conserved across species that harbor the p53 protein. Thus, a change in the zinc-binding motif of p53 may alter its stable conformational states. Hence, highlighting the significant role of His179 in the protein’s function and its ability to bind to the Zn^2+^ ion. The current report aims to identify key mutational hotspots from mapped genomic profiles and extrapolate our current understanding of the effects of numerous mutations on binding sites with the Zn^2+^ ion. H179Y, H179R, H179N, H179L, and H179D were the common mutations observed in the NSCLC subtypes, namely, LUAD and LUSC. The deleterious nature of the variants considered in the current study, as indicated by Predict-SNP results, underscores their significance in NSCLC subtypes.

Of the mutations considered in the current study, H179L results in a hydrophobic change at the 179th position, whereas the other mutations retain their hydrophilic nature at this position. Results of MDS for the H179L variant, indicate increased fluctuations in L1 and L2 regions of p53 protein. Figure 5 shows the residual interaction contact map of the L2 region for the first simulation run, highlighting the minimum average distance between residue pairs. Residues 10(R174)-15(His179) in particular, of the L2 region (Tyr163-Ile195), have shown significant average deviation. Figure 5E, which shows results for the H179L variant, shows increased residual distances between residues R174 (10)-179th position (15) (encircled in red). This indicates reduced intramolecular residual interactions and a more open and flexible conformation of the zinc-binding motifs in the L2 region of the protein, also showcased in Supplementary Figure S7. Contrastingly, dense interactions and reduced residual distances were observed for the H179N and H179D variants. Upon further examination of the distance between Zn and the 179th residue, the H179L variant showcased significant deviation in distance throughout the trajectory (Supplementary Table S2).

Furthermore, upon evaluating the KDE plots shown in Figure 6, a lower SASA value of 105 nm^2^ was observed at the epicenter in Figure 6E. A significantly weaker association is observed in the H179L mutant type, as evidenced by two epicenters in the KDE plot, suggesting a reduced correlation between solvent-exposed surface area and protein compactness. This reduced correlation indicates that the protein is losing its compact nature, resulting in a reduced solvent-exposed surface area (Nikolsky et al., 2023). Analysis of the secondary structures shows alterations in the coil content of the mutants compared to the wild type, highlighted in Supplementary Table S4. Additionally, the energy landscape of the protein conformations attained during the trajectory was evaluated and is showcased in Figure 7. The highest FEL value was observed for the H179L mutant (13.5 kJ mol-1), indicating that the protein adopts unstable, unfavorable conformations. Basin depths of global energy maxima observed for the protein conformations attained during the trajectory indicate relatively lower global energy maxima of 10.80 kJ mol^-1^ were observed for H179N, relative to 12–12.15 kJ mol^-1^ for the H179Y/R/D mutants. The interaction of the Zn^2+^ ion with the protein as a whole was also evaluated using MMPBSA calculations and distance measurements throughout the trajectory. The results of the one-ANOVA statistical test indicated significant alteration in Zn^2+^ - binding affinity for H179N and H179D variants, shown in Supplementary Table S5.

Studies have highlighted lung-enriched mutations (V157F, R158L, R175H) that cause a gain of function of p53 in cancer initiation, *via* similar structure-based *in silico* studies, which comment on aggregation-prone states and changes to the Rg and SASA properties of the p53 protein and its mutant types (Lei et al., 2021; 2022). The mechanism of pathogenicity of p53 variants also lies in the p53 protein’s inability to recognize the consensus DNA sequences for DNA repair. The L1 region of p53 is also known for the interaction of Lys120 with 06 and N7 of Gua^8^ by donating hydrogen bonds (Wright and Lim, 2007). A thorough interpretation of the RMSF graph shown in Figure 3B highlights the regions undergoing the highest deviation throughout the trajectory. Supplementary Figure S4-S6 showcase the trajectory deviations for L1, L2, and L3 regions, respectively. Significant fluctuations were observed in the L1 region for all mutants, moderate fluctuations in the L2 region, and minor fluctuations with a smoother trajectory in the L3 region, as shown in Table 4. Notable deviation was observed in the L1 region of the H179L variant, with a value of 0.422 ± 0.135 nm. Similarly, the least deviations in the L1 region, 0.359 ± 0.027 nm, were observed for the wildtype. Overall, MDS results obtained in the current study, indicate notable fluctuations in key regions, unstable conformation states and altered energy landscape of the p53 core domain due to His179 mutants. Increased fluctuation in L1 region, a key region for DNA recognition and binding, would hint towards unfavored binding to DNA sequences. A study on Y220C-p53 mutant variant highlighted compromised protein stability resulting from configurational changes in the L1 region (Lima et al., 2023). Studies have also attempted to reactivate mutant p53 using potential drug targets, specifically MQ, which bind to the L1/S3 pocket of the protein. However, due to the pronounced fluctuations and configurational changes observed in the L1 loop, it is challenging to dock and target small molecules to reactivate p53 mutants (Lambert et al., 2009; Wassman et al., 2013). Interaction dynamics between the L1 region and DNA of p53 for WT and His179 mutants is a key area of interest that remains unexplored and presents promising future scope.

Furthermore, LUAD and LUSC, despite being subtypes of NSCLC, show distinct tumor mutational burden and mutational frequencies (Nu Er Lan et al., 2024). LUAD-affected individuals were found to have more frequent and higher counts of genomic alterations in *MET*, *ALK*, and *ROS1* than LUSC-affected individuals (Caso et al., 2021; Nguyen et al., 2022). However, addressing both common and distinct mutational patterns in LUAD and LUSC will provide a holistic view for diagnosis to researchers and clinicians. Although the lack of clinical consequences of His179 substitutions in p53 on tumor suppression and DNA repair poses a limitation for the current study, it also provides a strong foundation for future prospective studies to build upon. However, the strength of the current study lies in the mechanistic insights into critical regions of p53 that undergo structural plasticity due to His179 substitutions, thereby providing researchers with the knowledge to devise therapeutic strategies to target p53 variants.

## 5 Conclusion

The current study evaluates the mutational profiles of individuals affected by NSCLC subtypes. It extrapolates the mutational findings to the proteome level by simulating the behavioral dynamics of the p53 protein in both apo and variant states. The synergistic interaction of Zn^2+^ with p53 protein is of utmost importance for the functioning of the p53 tetramer. H179Y, H179R, H179N, H179L, and H179D were the five mutations that were simulated and studied. Significant fluctuations were observed for all variants, as compared to the wild type. Simulations indicate increased deviation and flexibility of the L1 region in all mutants compared to the wild type, thereby hinting towards impaired DNA recognition and binding. Evaluation of conformational and energy landscapes indicates irregularities observed for all variants, as compared to the wildtype. Binding affinity analysis of the protein-Zn^2+^ complex was also carried out. Our report also highlights the scientific advantage of MDS over other computational tools in predicting and analyzing the effects of mutations on protein stability and structure. Studies evaluating the behavioral dynamics of mutant protein complexes help researchers understand the underlying mechanisms of action and identify druggable targets, thereby significantly improving the scope of drug discovery and drug repurposing.

## Data Availability

The original contributions presented in the study are included in the article/Supplementary Material, further inquiries can be directed to the corresponding author.
