# Supplementary material for: Integrated molecular dynamics elucidation of TP53 H179 zinc-binding variants: genomic and structural characterization across NSCLC subtypes
Source: Front Bioinform. 2026 Apr 10;6:1736501. doi: 10.3389/fbinf.2026.1736501 (PMC13106391; doi:10.3389/fbinf.2026.1736501)
Supplement: Supplementary file 4 [file Table1.docx]

**Supplementary Table 1:** List of mutations retrieved from TCGA database, for LUAD, LUSC and the common mutations observed amongst the 2 sub-types of NSCLC.

| Disease | List of Mutations |
| --- | --- |
| LUAD (359) | p.R157L, p.R273L, p.V157F, p.R273H, p.R175H, p.R248W, p.R282W, p.R248Q, p.G245V, p.R248L, p.G154V, p.R280T, p.Y163C, p.M237I, p.G245C, p.R273C, p.Y220C, p.H214R, p.R249S, p.A159P, p.G266R, p.C176F, p.G244C, p.Y234C, p.R249M, p.S215I, p.V272L, p.C277F, p.V272M, p.L194R, p.C242F, p.Y205C, p.R280I, p.G245S, p.G245D, p.H179R, p.R110L, p.E224D, p.G105C, p.R337L, p.V274F, p.C135F, p.V173L, p.V172F, p.C275F, p.Y236C, p.F270V, p.M246V, p.P278S, p.G199E, p.P152L, p.V216M, p.G266V, p.S241F, p.K132R, p.R249G, p.A159V, p.P250L, p.R282G, p.C238F, p.R248P, p.H179L, p.E258K, p.R283P, p.E285V, p.F134L, p.V143M, p.R156P, p.C275Y, p.R280K, p.R337C, p.R158P, p.P151S, p.G262V, p.H179Y, p.D281V, p.R110P, p.G334V, p.T155P, p.E285K, p.P278A, p.C176Y, p.P152S, p.A161T, p.P151A, p.G266E, p.N131I, p.Y163H, p.M246L, p.R248G, p.L194F, p.R213L, p.K132N, p.C135Y, p.K132E, p.V216L, p.C242Y, p.E286K, p.C141W, p.I332S, p.E180K, p.I255F, p.C238R, p.I195T, p.F113C, p.R273P, p.S241Y, p.H193R, p.R273S, p.A138V, p.R181P, p.S241T, p.T155I, p.R158C, p.T125K, p.R158H, p.E358Q, p.N247I, p.S127F, p.R273G, p.P72R, p.N239S, p.R280G, p.N239D, p.C275W, p.R267W, p.C242S, p.R267L, p.C238Y, p.G244D, p.R175L, p.R249T, p.C176S, p.S240G, p.P278T, p.G105D, p.P278L, p.R158G, p.P177R, p.A161V, p.R282P, p.R249W, p.D281E, p.G244S, p.M246K, p.R273Q, p.Q331H, p.K132Q, p.P190L, p.P151R, p.R213Q, p.S240F, p.R196P, p.G105V, p.M246I, p.K132M, p.Y236H, p.S215R, p.Y220S, p.Q144L, p.Y126C, p.S127Y, p.D281N, p.P278H, p.D259Y, p.I255T, p.H193D, p.E287D, p.C275S, p.Q136E, p.P152T, p.I251F, p.H168R, p.I232F, p.M243I, p.E271V, p.S241C, p.R342P, p.Y163D, p.E258D, p.P278R, p.V10I, p.A276D, p.N235D, p.R267P, p.E11Q, p.L130F, p.D281Y, p.G334W, p.L257V, p.R337P, p.E285Q, p.A276G, p.K139T, p.S116F, p.K139N, p.R175S, p.R174W, p.W146C, p.Y234H, p.V31I, p.R306P, p.D208V, p.V143A, p.C238S, p.V274D, p.C135R, p.K120E, p.A138S, p.R158F, p.E286Q, p.I332N, p.E271Q, p.T211I, p.N131K, p.Y163N, p.D281G, p.I254S, p.R202C, p.I254M, p.C242G, p.I254L, p.C229Y, p.R156L, p.C141R, p.I232N, p.A355T, p.R156G, p.R175G, p.T155N, p.F109V, p.I195N, p.E287Q, p.I195M, p.E286A, p.I195K, p.E285_E286delinsDK, p.I195F, p.E258V, p.I162S, p.V172D, p.I162F, p.D48H, p.H233Y, p.M246T, p.R280S, p.R213G, p.H214L, p.D186H, p.H193Y, p.M237V, p.R110C, p.C242W, p.H193P, p.L348F, p.H193N, p.C238G, p.H193L, p.A138P, p.Y126N, p.C141Y, p.Q192_H193delinsHY, p.L145R, p.Q144P, p.A79T, p.H179Q, p.A276S, p.H179N, p.L137Q, p.T155A, p.L111Q, p.H179D, p.V203L, p.H168Y, p.F109C, p.Q144K, p.E298V, p.H168L, p.P151T, p.Q144H, p.S215N, p.V272K, p.V173A, p.G334A, p.S215G, p.G325V, p.P128A, p.P77L, p.E271K, p.G266T, p.E258Q, p.T102P, p.S127P, p.P71L, p.S127C, p.P60S, p.D49V, p.S94L, p.D391N, p.S269G, p.S106R, p.G245R, p.D281H, p.G245L, p.S106I, p.S261G, p.D228H, p.V272G, p.D208N, p.G245A, p.D148E, p.G244V, p.V274L, p.V272E, p.M237K, p.G244F, p.M169I, p.V218L, p.M160I, p.P250S, p.M133I, p.G226D, p.C238W, p.G226C, p.L344R, p.Y126D, p.L289P, p.G187S, p.C182Y, p.G187R, p.C176W, p.P250F, p.V147G, p.G154S, p.R337G, p.G154I, p.C141F, p.G154D, p.C135T, p.P190S, p.L145Q, p.P190R, p.A70V, p.S241A, p.A347D, p.G105A, p.A276P, p.Y205H, p.L145P, p.F270L, p.L130V, p.F270C, p.A159S, p.R267G, p.K291T, p.F113V, p.K164E, p.F113S, p.L201F |
| LUSC (254) | p.V157F, p.R158L, p.R273L, p.R248L, p.Y220C, p.R249S, p.R282W, p.Y163C, p.R175H, p.R248W, p.G245C, p.R273H, p.C242F, p.H179R, p.M237I, p.Y234C, p.T155P, p.G245V, p.R267P, p.G154V, p.R337L, p.C176Y, p.H179Y, p.R273C, p.Y205C, p.R181P, p.R248Q, p.V173M, p.R283P, p.R249M, p.H214R, p.P278L, p.R273P, p.R158P, p.K132R, p.Y163H, p.C141Y, p.C242Y, p.R110L, p.Y236C, p.L194R, p.R280I, p.G262V, p.C176F, p.P278R, p.H193R, p.H179L, p.A159V, p.R175G, p.N239D, p.R282G, p.R196P, p.E286K, p.G244C, p.D281H, p.G245D, p.E271K, p.G266R, p.C277F, p.G266V, p.R248P, p.C238F, p.R280K, p.C242S, p.E258K, p.R110P, p.E285K, p.C275F, p.C275Y, p.P278S, p.D281Y, p.G245S, p.R337C, p.G266E, p.D281E, p.G266L, p.G244V, p.H168P, p.T125P, p.H193L, p.V272M, p.C176S, p.D281V, p.H296L, p.R280G, p.I162F, p.A276G, p.I195T, p.S106R, p.K164Q, p.T284P, p.C238S, p.E285V, p.C238Y, p.D259Y, p.N239S, p.R249W, p.P151H, p.R273G, p.P151S, p.R273S, p.P190L, p.E224D, p.P250L, p.R280S, p.Y234D, p.F270I, p.Q331H, p.E258V, p.R156H, p.S241F, p.R158G, p.T256P, p.A161T, p.V147A, p.R174K, p.V173L, p.R175L, p.V272G, p.F270C, p.D208V, p.C135Y, p.I251F, p.T155N, p.E258_D259delinsDH, p.M246T, p.V197G, p.N131I, p.K132N, p.N210H, p.S215R, p.A129V, p.L252P, p.A159D, p.Y107Q, p.N239T, p.I255N, p.N263H, p.K164E, p.N268H, p.K320E, p.N288S, p.K370Q, p.P142A, p.C141W, p.A159P, p.V172G, p.P151R, p.H179N, p.H193D, p.E294Q, p.P152L, p.G244S, p.P153A, p.D49H, p.P153H, p.K132T, p.P177L, p.R282P, p.G105C, p.K292N, p.P190T, p.S149T, p.H193N, p.S240C, p.P250S, p.L111Q, p.P278A, p.T230N, p.P278H, p.V143E, p.A161D, p.V157G, p.C242G, p.L348F, p.F109C, p.V216E, p.H193P, p.M237K, p.Y234S, p.C135W, p.Y236S, p.M237T, p.Q144P, p.R267G, p.G293E, p.I255F, p.G105V, p.I255V, p.G154S, p.E180D, p.H193Y, p.G105R, p.R156P, p.K139N, p.Y220S, p.H179D, p.R158F, p.R282Q, p.A138V, p.K291R, p.R158H, p.K319N, p.F270L, p.S127F, p.A276D, p.S215G, p.H296Y, p.S215T, p.R174M, p.S241C, p.R174W, p.T125M, p.C275W, p.T155I, p.G187V, p.G245R, p.H168Y, p.L111R, p.R181C, p.V143A, p.I195F, p.V143L, p.G244D, p.L265P, p.R209K, p.V172F, p.R209S, p.V173A, p.R213G, p.M133R, p.R248G, p.V197L, p.I195M, p.V216M, p.G244R, p.V272L, p.D228N, p.V31I, p.D259V, p.Y126D, p.R249G, p.E286V, p.I195S, p.E336G, p.H178P, p.R249T, p.R158C, p.Y234H, p.P72A, p.F113C, p.Q136E, p.F134L, p.Q136H |
| COMMON (176) | p.V157F, p.R158L, p.R273L, p.R248L, p.Y220C, p.R249S, p.R282W, p.Y163C, p.R175H, p.R248W, p.G245C, p.R273H, p.C242F, p.H179R, p.M237I, p.Y234C, p.T155P, p.G245V, p.R267P, p.G154V, p.R337L, p.C176Y, p.H179Y, p.R273C, p.Y205C, p.R181P, p.R248Q, p.R283P, p.R249M, p.H214R, p.P278L, p.R273P, p.R158P, p.K132R, p.Y163H, p.C141Y, p.C242Y, p.R110L, p.Y236C, p.L194R, p.R280I, p.G262V, p.C176F, p.P278R, p.H193R, p.H179L, p.A159V, p.R175G, p.N239D, p.R282G, p.R196P, p.E286K, p.G244C, p.D281H, p.G245D, p.E271K, p.G266R, p.C277F, p.G266V, p.R248P, p.C238F, p.R280K, p.C242S, p.E258K, p.R110P, p.E285K, p.C275F, p.C275Y, p.P278S, p.D281Y, p.G245S, p.R337C, p.G266E, p.D281E, p.G244V, p.H193L, p.V272M, p.C176S, p.D281V, p.R280G, p.I162F, p.A276G, p.I195T, p.S106R, p.C238S, p.E285V, p.C238Y, p.D259Y, p.N239S, p.R249W, p.R273G, p.P151S, p.R273S, p.P190L, p.E224D, p.P250L, p.R280S, p.Q331H, p.E258V, p.S241F, p.R158G, p.A161T, p.V173L, p.R175L, p.V272G, p.F270C, p.D208V, p.C135Y, p.I251F, p.T155N, p.M246T, p.N131I, p.K132N, p.S215R, p.K164E, p.C141W, p.A159P, p.P151R, p.H179N, p.H193D, p.P152L, p.G244S, p.R282P, p.G105C, p.H193N, p.P250S, p.L111Q, p.P278A, p.P278H, p.C242G, p.L348F, p.F109C, p.H193P, p.M237K, p.Q144P, p.R267G, p.I255F, p.G105V, p.G154S, p.H193Y, p.R156P, p.K139N, p.Y220S, p.H179D, p.R158F, p.A138V, p.R158H, p.F270L, p.S127F, p.A276D, p.S215G, p.S241C, p.R174W, p.C275W, p.T155I, p.G245R, p.H168Y, p.V143A, p.I195F, p.G244D, p.V172F, p.V173A, p.R213G, p.R248G, p.I195M, p.V216M, p.V272L, p.V31I, p.Y126D, p.R249G, p.R249T, p.R158C, p.Y234H, p.F113C, p.Q136E, p.F134L |
