# Supplementary material for: Integrated molecular dynamics elucidation of TP53 H179 zinc-binding variants: genomic and structural characterization across NSCLC subtypes
Source: Front Bioinform. 2026 Apr 10;6:1736501. doi: 10.3389/fbinf.2026.1736501 (PMC13106391; doi:10.3389/fbinf.2026.1736501)

**Supplementary Figure S3:** RMSD plot featuring the RMSD Standard Error along with the rolling average of the triplicate trajectories for (A) Wildtype; (B) H179Y variant; (C) H179R variant; (D) H179N variant; (E) H179L variant; (F) H179D variant. The average fluctuation has been depicted in black, with the standard error of margin showcased in orange.


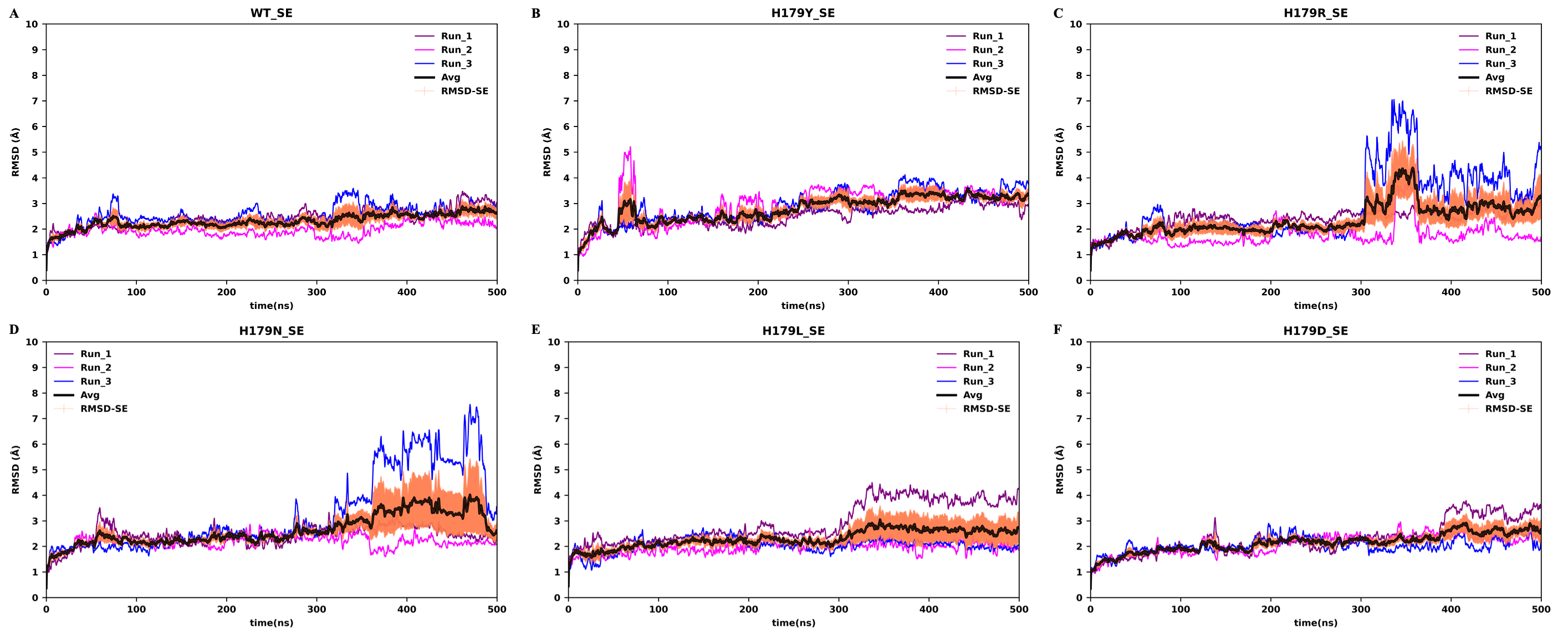

Supplement: Supplementary file 5 [file DataSheet3.docx]
