# Supplementary material for: Integrated molecular dynamics elucidation of TP53 H179 zinc-binding variants: genomic and structural characterization across NSCLC subtypes
Source: Front Bioinform. 2026 Apr 10;6:1736501. doi: 10.3389/fbinf.2026.1736501 (PMC13106391; doi:10.3389/fbinf.2026.1736501)
Supplement: Supplementary file 7 [file Table2.docx]

**Supplementary Table 2:** Distances measured with Zn^2+^ to 179^th^ residue in each complex at every 100ns interval.

| Timestamp | WT | H179Y | H179R | H179N | H179L | H179D |
| --- | --- | --- | --- | --- | --- | --- |
| Distance at 0 ns (nm) | 0.45 | 0.39 | 0.33 | 0.21 | 0.4 | 0.2 |
| Distance at 100 ns (nm) | 0.4 | 0.46 | 0.47 | 0.21 | 0.44 | 0.2 |
| Distance at 200 ns (nm) | 0.42 | 0.5 | 0.47 | 0.21 | 0.49 | 0.2 |
| Distance at 300 ns (nm) | 0.41 | 0.5 | 0.45 | 0.21 | 0.44 | 0.2 |
| Distance at 400 ns (nm) | 0.43 | 0.46 | 0.42 | 0.21 | 0.51 | 0.19 |
| Distance at 500 ns (nm) | 0.42 | 0.47 | 0.44 | 0.2 | 0.52 | 0.2 |
