# Supplementary material for: Integrated molecular dynamics elucidation of TP53 H179 zinc-binding variants: genomic and structural characterization across NSCLC subtypes
Source: Front Bioinform. 2026 Apr 10;6:1736501. doi: 10.3389/fbinf.2026.1736501 (PMC13106391; doi:10.3389/fbinf.2026.1736501)
Supplement: Supplementary file 8 [file Table5.docx]

**Supplementary Table 5:** Results of the one-ANOVA statistical test for triplicate trajectories of MMPBSA Zinc-binding affinity.

| Group1 | Group2 | Mean Diff | p-adj | lower | upper | Significant |
| --- | --- | --- | --- | --- | --- | --- |
| H179D | WT | 61.4833 | 0.0005 | 28.1428 | 94.8238 | TRUE |
| H179L | WT | 4.4 | 0.9973 | -28.9405 | 37.7405 | FALSE |
| H179N | WT | 42.68 | 0.0102 | 9.3395 | 76.0205 | TRUE |
| H179R | WT | 24.2233 | 0.2168 | -9.1172 | 57.5638 | FALSE |
| H179Y | WT | 2.7633 | 0.9997 | -30.5772 | 36.1038 | FALSE |
