# Supplementary material for: Integrated molecular dynamics elucidation of TP53 H179 zinc-binding variants: genomic and structural characterization across NSCLC subtypes
Source: Front Bioinform. 2026 Apr 10;6:1736501. doi: 10.3389/fbinf.2026.1736501 (PMC13106391; doi:10.3389/fbinf.2026.1736501)
Supplement: Supplementary file 9 [file Table3.docx]

**Supplementary Table 3:** Case counts for each variant substitution for LUAD and LUSC affected individuals.

| Variant | LUAD | LUSC |
| --- | --- | --- |
| H179Y (c.535C>T) | 4 | 6 |
| H179R (c.536A>G) | 9 | 9 |
| H179N (c.535C>A) | 1 | 1 |
| H179L (c.536A>T) | 6 | 4 |
| H179D (c.535C>G) | 1 | 1 |
