# Supplementary material for: Integrated molecular dynamics elucidation of TP53 H179 zinc-binding variants: genomic and structural characterization across NSCLC subtypes
Source: Front Bioinform. 2026 Apr 10;6:1736501. doi: 10.3389/fbinf.2026.1736501 (PMC13106391; doi:10.3389/fbinf.2026.1736501)

**Supplementary Figure S2:** Results of DSSP analysis wherein C stands for Frequency of Coil, H represents Frequency of Helix and E highlights the frequency of Sheets for. The major deviations in coils have been highlighted in hollow red circle. (A) Wildtype; (B) H179Y variant; (C) H179R variant; (D) H179N variant; (E) H179L variant; (F) H179D variant. Changes to B-sheet regions and coils were observed across the variants.


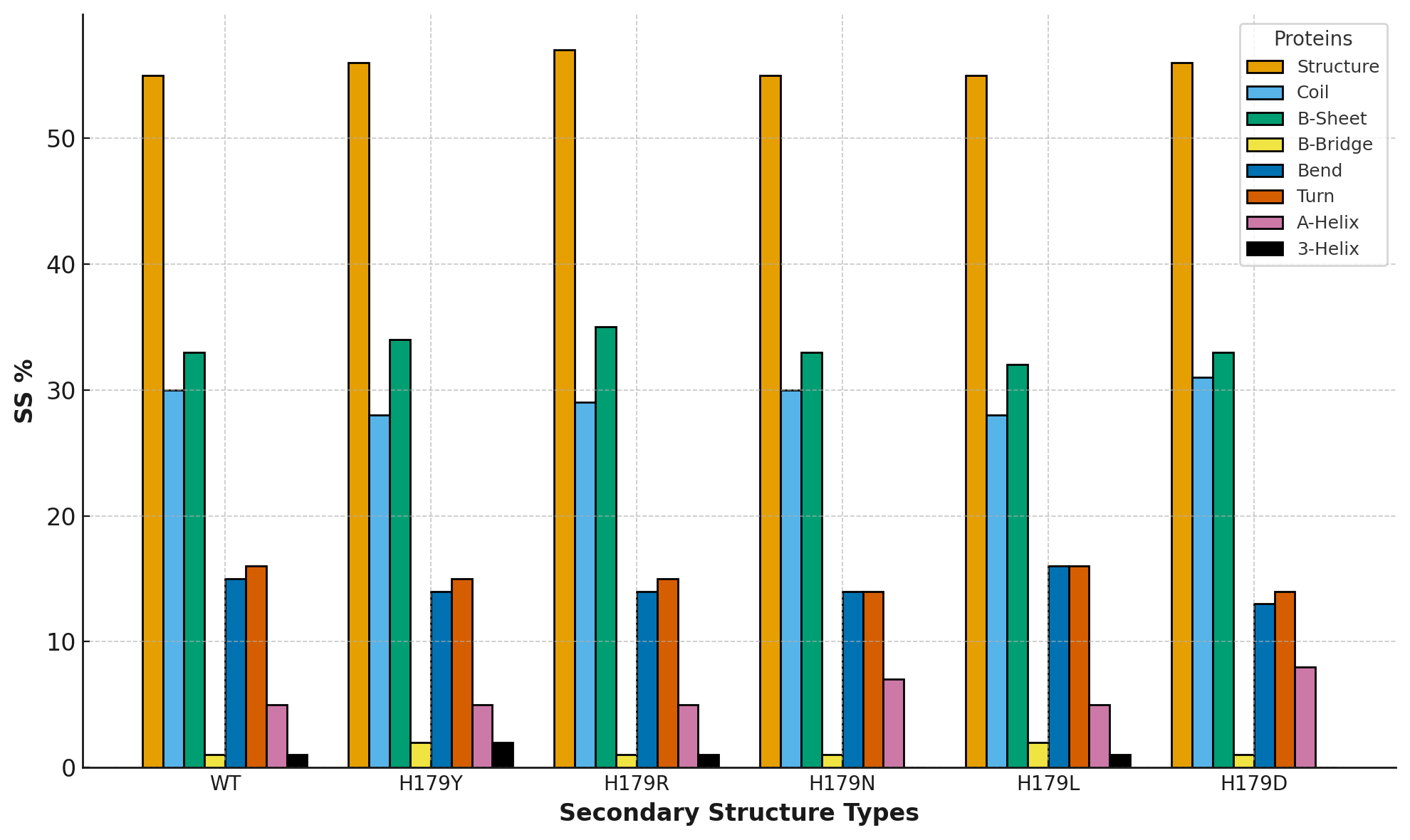

Supplement: Supplementary file 10 [file DataSheet2.docx]
