# Supplementary material for: Integrated molecular dynamics elucidation of TP53 H179 zinc-binding variants: genomic and structural characterization across NSCLC subtypes
Source: Front Bioinform. 2026 Apr 10;6:1736501. doi: 10.3389/fbinf.2026.1736501 (PMC13106391; doi:10.3389/fbinf.2026.1736501)
Supplement: Supplementary file 11 [file Table4.docx]

**Supplementary Table 4:** Percentage metrics observed for the secondary structure elements of p53.

| Variant | Structure (%) | Coil (%) | B-Sheet (%) | B-Bridge (%) | Bend (%) | Turn (%) | A-Helix (%) | 3-Helix (%) |
| --- | --- | --- | --- | --- | --- | --- | --- | --- |
| WT | 55 | 30 | 33 | 1 | 15 | 16 | 5 | 1 |
| H179Y | 56 | 28 | 34 | 2 | 14 | 15 | 5 | 2 |
| H179R | 57 | 29 | 35 | 1 | 14 | 15 | 5 | 1 |
| H179N | 55 | 30 | 33 | 1 | 14 | 14 | 7 | 0 |
| H179L | 55 | 28 | 32 | 2 | 16 | 16 | 5 | 1 |
| H179D | 56 | 31 | 33 | 1 | 13 | 14 | 8 | 0 |
