# Supplementary material for: Integrated molecular dynamics elucidation of TP53 H179 zinc-binding variants: genomic and structural characterization across NSCLC subtypes
Source: Front Bioinform. 2026 Apr 10;6:1736501. doi: 10.3389/fbinf.2026.1736501 (PMC13106391; doi:10.3389/fbinf.2026.1736501)

**Supplementary Figure S1:** Variant distribution plotted in the form of a dashboard highlighting numerous variant distribution metrics for (A) LUAD; (B) LUSC. C > A was the predominant SNP observed upon studying the mutational profiles of LUAD and LUSC. A median of 158 genomic variants were observed for a LUAD affected individual, while for a LUSC affected individual a median value of 191 genomic variants was observed.


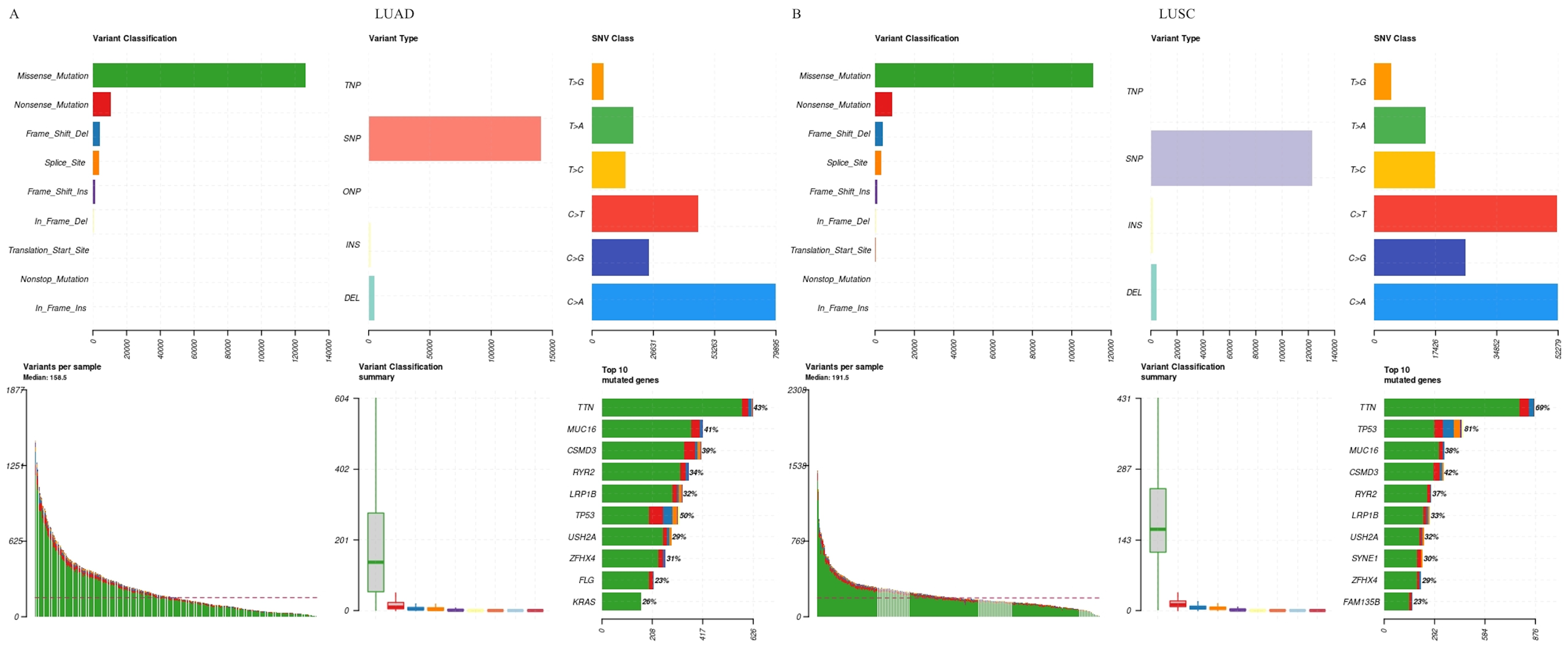

Supplement: Supplementary file 12 [file DataSheet1.docx]
